# Using Quantitative Bias Analysis to Adjust for Misclassification of COVID‐19 Outcomes: An Applied Example of Inhaled Corticosteroids and COVID‐19 Outcomes

**DOI:** 10.1002/pds.70086

**Published:** 2025-01-07

**Authors:** Marleen Bokern, Christopher T. Rentsch, Jennifer K. Quint, Jacob Hunnicutt, Ian Douglas, Anna Schultze

**Affiliations:** ^1^ Department of Non‐Communicable Disease Epidemiology London School of Hygiene and Tropical Medicine London UK; ^2^ Faculty of Medicine, National Heart & Lung Institute, Imperial College London London UK; ^3^ Boehringer Ingelheim Pharmaceuticals, Inc. Ridgefield Connecticut USA

**Keywords:** COVID‐19, misclassification, pharmacoepidemiology, quantitative bias analysis, respiratory epidemiology

## Abstract

**Background:**

During the pandemic, there was concern that underascertainment of COVID‐19 outcomes may impact treatment effect estimation in pharmacoepidemiologic studies. We assessed the impact of outcome misclassification on the association between inhaled corticosteroids (ICS) and COVID‐19 hospitalisation and death in the United Kingdom during the first pandemic wave using probabilistic bias analysis (PBA).

**Methods:**

Using data from the Clinical Practice Research Datalink Aurum, we defined a cohort with chronic obstructive pulmonary disease (COPD) on 1 March 2020. We compared the risk of COVID‐19 hospitalisation and death among users of ICS/long‐acting β‐agonist (LABA) and users of LABA/LAMA using inverse probability of treatment weighted (IPTW) logistic regression. We used PBA to assess the impact of non‐differential outcome misclassification. We assigned beta distributions to sensitivity and specificity and sampled from these 100 000 times for summary‐level and 10 000 times for record‐level PBA. Using these values, we simulated outcomes and applied IPTW logistic regression to adjust for confounding and misclassification. Sensitivity analyses excluded ICS + LABA + LAMA (triple therapy) users.

**Results:**

Among 161 411 patients with COPD, ICS users had increased odds of COVID‐19 hospitalisations and death compared with LABA/LAMA users (OR for COVID‐19 hospitalisation 1.59 (95% CI 1.31–1.92); OR for COVID‐19 death 1.63 (95% CI 1.26–2.11)). After IPTW and exclusion of people using triple therapy, ORs moved towards the null. All implementations of QBA, both record‐ and summary‐level PBA, modestly shifted the ORs away from the null and increased uncertainty.

**Conclusions:**

We observed increased risks of COVID‐19 hospitalisation and death among ICS users compared to LABA/LAMA users. Outcome misclassification was unlikely to change the conclusions of the study, but confounding by indication remains a concern.

**Summary**

- COVID‐19 outcomes may have been at risk of misclassification early during the COVID‐19 pandemic.
- No pharmacoepidemiological studies have evaluated the impact of such misclassification on their findings.
- We used several forms of quantitative bias analysis to adjust for outcome misclassification in an analysis of inhaled corticosteroids and COVID‐19 hospitalisation and death.
- We found that all methods shifted effect estimates away from the null, albeit to a limited extent.
- This study provides reassurance that even relatively substantial outcome misclassification would not have changed the study conclusions and demonstrates how to correct for cause‐specific outcome misclassification in epidemiological studies.

## 1 Introduction

At the beginning of the pandemic, many pharmacoepidemiologic studies were conducted to investigate the effects of existing medications on COVID‐19 outcomes. These studies may have been affected by biases, leading to potentially differing results compared to randomised controlled trials (RCTs).

The role of confounding was considered in detail in these studies [1, 2, 3, 4], but the potential role of misclassification received less attention. Outcome misclassification is likely to have occurred during the early use of new International Classification of Disease 10th revision (ICD‐10) codes denoting COVID‐19 diagnoses [5, 6]. In particular, there was concern that COVID‐19 mortality would be underestimated when considering official reports of COVID‐19 deaths alongside excess all‐cause mortality [6]. Inhaled corticosteroids (ICS), anti‐inflammatory drugs widely used to treat COPD [7], were one of the medications investigated as potential treatments for COVID‐19 due to their immunosuppressant effects [8, 9], which could have different consequences at different stages of infection [10]. Observational studies found inconsistent results, but RCTs subsequently found a protective effect of one inhaled ICS, budesonide, on severe COVID‐19 outcomes [11, 12].

This study assesses the potential impact of outcome misclassification in an analysis of ICS/LABA (long‐acting β‐agonist) use compared with LABA/LAMA (long‐acting muscarinic antagonist) use on the risk of COVID‐19 hospitalisation or death during the first wave of COVID‐19 in the United Kingdom (March–August 2020), using three different methods of quantitative bias analysis (QBA).

## 2 Methods

The study protocol was registered on ENCEPP EU PAS (Register Number: 47885), and we completed the RECORD‐PE checklist (Table S1) [13].

### 2.1 Study Design

#### 2.1.1 Data Source

This study used routinely collected data from primary care in the United Kingdom in the Clinical Practice Research Datalink (CPRD) Aurum. CPRD Aurum includes data on 41 million patients (May 2022 build) from > 1300 general practices [14] and is representative of the English population [15].

CPRD Aurum was linked to Hospital Episode Statistics (HES) Admitted Patient Care (APC) and Office for National Statistics (ONS) Death Registry [15, 16]. HES APC holds information on all in‐patient contacts at NHS hospitals in England [16, 17]. The ONS Death Registry contains information on deaths occurring in England and Wales, including the cause of death documented using ICD‐10 codes [17, 18]. Data were linked to Index of Multiple Deprivation (IMD), a postcode‐level indicator of socioeconomic status.

#### 2.1.2 Study Population

We defined a cohort of people with COPD before 1 March 2020 (i.e., the index date) based on a validated algorithm [19]. Patients had to be alive, aged ≥ 35, registered in CPRD Aurum on 1 March 2020 and have ≥ 12 months' continuous registration before the index date. In the main analysis, we excluded people with asthma within 3 years before the index date, leukotriene receptor antagonist use within 4 months before the index date as this indicates asthma or other chronic respiratory disease at any point before the index date. Patients were followed up until death, deregistration or 31 August 2020, whichever came first. If death was registered in ONS, that date was considered the date of death. If death was missing in ONS but registered in CPRD, we used the date recorded in CPRD as the date of death. A study diagram [20] is provided in Figure S1.

##### 2.1.2.1 Exposure

Treatment episodes were estimated based on the prescription issue date and information on the intended duration, prescribed amount and dosage (Method S1).

We used the derived exposure start and end dates to identify people using ICS/LABA or LABA/LAMA on the index date, either as combined or separate inhalers. ICS/LABA was the exposure of interest, and LABA/LAMA was the active comparator. People using ICS + LABA + LAMA (henceforth referred to as triple therapy) were included in the ICS/LABA group but were excluded in a sensitivity analysis.

##### 2.1.2.2 Outcome

The outcomes were (i) hospitalisation with a primary diagnosis code for COVID‐19 in HES APC, and (ii) death with a code for COVID‐19 as a cause of death anywhere on the death certificate in the ONS Death Registry. Diagnostic codes for COVID‐19 were ICD‐10 U07.1 and U07.2.

#### 2.1.3 Covariates

Results were adjusted for the following baseline covariates: age, gender, BMI (most recent and within the previous 10 years, categorised as underweight (< 18.5), normal (18.5–24.9), overweight (25–29.9) or obese (≥ 30)), smoking (current vs. former), ethnicity, cancer, diabetes, chronic kidney disease, cardiovascular disease, hypertension, asthma (not within the past 3 years), immunosuppression, receipt of influenza vaccine (past year), receipt of pneumococcal vaccine (past 5 years), IMD quintile and COPD exacerbation in the past year. COPD exacerbations were identified based on a validated algorithm [21].

### 2.2 Statistical Analyses

Cohort characteristics were summarised using descriptive statistics by exposure group. There were missing data for BMI, ethnicity and IMD. Missing BMI values were assumed to be normal BMI [22]. Missing ethnicity and IMD were considered separate categories.

We estimated propensity scores (PSs) and used inverse probability of treatment weighting (IPTW) to estimate the average treatment effect (ATE), adjusting for potential confounders. PSs were estimated using logistic regression, including the covariates listed above. Weights were calculated as $\frac{1}{\mathrm{ps}}$ (ICS) and $\frac{1}{1-\mathrm{ps}}$ (LABA/LAMA), where the PS is the probability of receiving ICS. Overlap of the PSs across treatment groups was assessed graphically and by summarising by treatment group. PSs were trimmed to the region of common support [23].

Logistic regression was used to estimate odds ratios (ORs) and 95% confidence intervals (CIs). This was done to make estimates comparable across analyses, as simple bias analysis (SBA) and summary‐level PBA are conducted based on 2 × 2 tables, generating relative risks or ORs as relative effect estimates. Although we anticipated this to have minimal impact on findings as the follow‐up time was short and censoring was rare, we additionally conducted Cox regression where possible (analyses without QBA and record‐level PBA).

#### 2.2.1 QBA

##### 2.2.1.1 QBA for Outcome Misclassification

We used SBA and PBA [24] to investigate the potential impact of outcome misclassification using methods based on Fox et al. [25]. We used estimates of sensitivity and specificity as bias parameters to describe assumptions about the accuracy of COVID‐19 hospitalisation in HES and COVID‐19 death in ONS. We expected that COVID‐19 hospitalisations would have been ascertained with greater accuracy than COVID‐19 deaths. For both outcomes, we assumed outcome misclassification was non‐differential with respect to exposure, as hospitalisations and deaths would have been coded without exposure. This assumes exposure is not associated with characteristics that may predict outcome validity. This assumption was evaluated in sensitivity analyses of differential misclassification. We assumed occurrences and dates recorded in HES and ONS to be correct and corrected only for misclassified causes of hospitalisations and deaths. Therefore, correction for outcome misclassification was conducted only among people who were hospitalised or died of any cause. This involved conducting QBA steps among patients hospitalised or who died; patients without hospitalisation or death were subsequently included to calculate the effect estimates in the entire population. Code illustrating these steps is available on our GitHub repository (https://github.com/bokern/ics_covid).

Initially, we conducted SBA using available tools [26], using best estimates of sensitivity and specificity. We then performed summary‐ and record‐level PBA using Monte Carlo sampling of bias parameter values from prespecified distributions for both outcomes, generating a point estimate and 95% simulation interval (SI).

#### 2.2.2 Bias Parameter Values and Distributions

##### 2.2.2.1 Hospitalisations

In the absence of evidence from the United Kingdom, values for sensitivity and specificity of hospital diagnoses were based on validation studies from North America [27, 28] and bounds given by the data. Kadri et al. estimated a sensitivity = 98.0% and a specificity = 99.0% in US administrative data during April and May 2020 [27]. A validation study from Canada between March 2020 and February 2021 estimated a sensitivity = 82.5% [28]. We therefore estimated that sensitivity would have a median of 0.90 and lie between 0.80 and 0.96 (Table 1). As only 5% of hospitalised patients had a COVID‐19 hospitalisation, the proportion of false‐positive COVID‐19 hospitalisations could not exceed 5%, so specificity was > 95%. We assigned parameter values to beta distributions based on the mean and variance of the target distributions [29]. For sensitivity, we parameterised a beta distribution with median = 0.90, 2.5th percentile = 0.80 and 97.5th percentile = 0.96 (α = 47.7, β = 5.5). For specificity, we used a beta distribution with median = 0.98, 2.5th percentile = 0.95, 97.5th percentile = 0.999 (α = 1, β = 1, transformed to have a lower bound of 0.95). These distributions resulted in negative cell counts in the 2 × 2 table in 28% of iterations, indicating incompatibility with the data [29]. As negative cell counts were driven by false positives, we subsequently increased the lower bound of the specificity distribution to 0.97 to obtain plausible results.

**TABLE 1:** Decisions related to the implementation of quantitative bias analysis.

	Hospitalisation	Death
Bias parameters	Sensitivity and specificity	Sensitivity and specificity
Simple bias analysis		
Values	Se = 90.5%, Sp = 96.0%	Se = 72.2%, Sp = 96.6%
Source/rationale	Validation studies from the United States and Canada [27, 28, 30]. Bounds given by data.	Data on excess deaths in the United Kingdom (March–May 2020) [31, 32]
Differential with respect to exposure status?	No	No
Probabilistic bias analysis		
Type of distribution	Beta	Beta
Values (distribution)	Se ~ beta (47.7, 5.5) Sp = 0.95 + 0.05X, where X ~ beta (1, 1) Sp = 0.97 + 0.03X, where X ~ beta (1,1)	Se ~ beta (39.6, 15.4) Sp ~ beta (91.2, 3.9)
Source/rationale	Assumed that the Se would lie within ca. 10% on either side of the point estimate. Our data suggested that the Sp had a lower bound of 95%.	Assumed that Se would lie within ca. 10% on either side of the point estimate, and that Sp would lie roughly 4% on either side of the point estimate.
Number of samples (summary level)	100 000	100 000
Number of samples (record level)	10 000	10 000
Correlations of distributions	None	None

##### 2.2.2.2 Deaths

Data on excess deaths [31] and reported COVID‐19 deaths in England and Wales [32] were used to inform estimates of sensitivity and specificity (Method S2). Assuming all excess deaths between 1 March 2020 and 7 May 2020 were COVID‐19 deaths, we estimated a sensitivity = 72.2% and specificity = 96.6% among those who died of any cause (Table 1). We assumed that sensitivity and specificity estimates would lie within a 10% range of the point estimate, and parameterised a beta distribution for sensitivity with values median = 0.72, 2.5th percentile = 0.60 and 97.5th percentile = 0.83 (α = 39.6, β = 15.4). For specificity, we used a beta distribution with median = 0.96, 2.5th percentile = 0.91 and 97.5th percentile = 0.99 (α = 91.2, β = 3.9). Graphs of bias parameter distributions are in Figures S6–S11.

##### 2.2.2.3 QBA

We constructed 2 × 2 exposure‐outcome tables for the relevant population by restricting to people who were hospitalised or died of any cause. Patients with multiple hospitalisations were counted as having a COVID‐19 hospitalisation if any were recorded as due to COVID‐19. If none were recorded as due to COVID‐19, we only counted that patient once.

Using available tools [26], we applied the median of bias parameters (Se = 90.5% and Sp = 96.0% for COVID‐19 hospitalisations; Se = 72.2% and Sp = 96.6% for COVID‐19 deaths) to conduct SBA. SBA results in a single point estimate, but no estimate of uncertainty. Estimating the standard error from the adjusted 2 × 2 table would underestimate the true uncertainty as it would not reflect uncertainty introduced by the estimation of bias parameters and would therefore not be valid.

For summary‐level PBA, we sampled from the specified distributions 100 000 times and applied the sampled values to correct the 2 × 2 tables. For record‐level correction, we sampled from each distribution 10 000 times and corrected the outcome for each individual record [25]. Initially, sampled sensitivity and specificity were applied to the 2 × 2 table exposure‐outcome to estimate outcome prevalence in the exposure groups. From the estimated prevalences, sensitivity and specificity, we estimated positive and negative predictive values (PPV and NPV), which were again used to adjust the 2 × 2 table. For record‐level PBA, we conducted Bernoulli trials for each patient to simulate the outcome, using the estimated PPV and NPV for each combination of exposure and observed outcome. This step is omitted in summary‐level PBA.

After each iteration, we added back in patients without hospitalisations or deaths to conduct unweighted and IPT–weighted logistic regression, generating bias‐adjusted ORs with 95% SIs. As patients could have multiple hospitalisations, we simulated potential outcome misclassification for each hospitalisation and selected the first one simulated as being due to COVID‐19 to determine the outcomes.

A full description of the methods, including equations, is provided in Methods S3–S5.

##### 2.2.2.4 Sensitivity Analyses

To improve comparability of the treatment groups, we excluded people using triple therapy as we expected they would be sicker than those using dual therapy. To assess the sensitivity of the results to individual values of bias parameters, we conducted summary‐level PBA steps for individual values of sensitivity and specificity to generate the point estimate with a 95% SI for both outcomes. For COVID‐19 deaths, we additionally simulated outcome misclassification differential with respect to exposure by conducting the steps for summary‐level PBA, but for individual values of sensitivity among the ICS and LABA/LAMA groups with specificity set at 0.97. We conducted several other sensitivity analyses described in Method S6.

Data were managed using Stata Version 17.0 [33], and analysis was conducted using R (Version 4.3.3) [34]. Code lists and data management and analysis code are available on our GitHub repository (https://github.com/bokern/ics_covid).

## 3 Results

### 3.1 Description of the Study Population

The cohort included 161 411 patients with COPD (Figure S2). Of these, 56 059 (35.4%) were using ICS/LABA at baseline, and 22 319 (13.81%) were using LABA/LAMA. Median follow‐up was 183 days. The proportion of patients censored for any reason was 6.5% for COVID‐19 hospitalisations and 6.1% for COVID‐19 deaths (Table S2).

Cohorts were similar in all measured covariates apart from the proportion of people with a history of asthma and COPD exacerbations in the past year (Table 2). After IPT weighting, treatment groups were balanced on all covariates.

**TABLE 2:** Baseline demographic and clinical characteristics of patients in the cohort before and after inverse probability of treatment weighting.

	Before weighting			After inverse probability of treatment weighting		
	ICS *N* = 56 059	LABA/LAMA *N* = 22 319	SMD	ICS *N* = 56 051	LABA/LAMA *N* = 22 324	SMD
Age						
Mean (SD)	71.3 (10.5)	70.8 (10.2)	0.0480	71.18 (10.46)	71.19 (10.30)	−0.00170
Median (25%–75%)	71.7 (64.7–78.7)	71.7 (63.7–77.7)		71.67 (63.67–78.67)	71.67 (64.67–78.67)	
Gender						
Male	29 830 (53%)	12 245 (55%)		30 094 (54%)	12 036 (54%)	
Female	26 229 (47%)	10 074 (45%)	0.0166	25 957 (46%)	10 287 (46%)	0.00226
BMI						
Underweight (< 18.5)	3142 (5.6%)	970 (4.3%)	0.0126	17 928 (32%)	7112 (32%)	0.000344
Normal (18.5–24.9)	18 163 (32%)	6926 (31%)	0.0136	2936 (5.2%)	1162 (5.2%)	0.00126
Overweight (25–29.9)	17 349 (31%)	7172 (32%)	−0.0118	17 529 (31%)	6979 (31%)	9.41E‐05
Obese (> = 30)	17 405 (31%)	7251 (32%)	−0.0144	17 658 (32%)	7071 (32%)	−0.00170
Ethnicity						
White	49 390 (88%)	19 584 (88%)	0.00370	49 334 (88%)	19 648 (88%)	0.0
South Asian	742 (1.3%)	197 (0.9%)	0.00423	665 (1.2%)	280 (1.3%)	−0.000678
Black	351 (0.6%)	130 (0.6%)	0.000438	345 (0.6%)	140 (0.6%)	−9.71E‐05
Mixed	142 (0.3%)	49 (0.2%)	0.000338	138 (0.2%)	61 (0.3%)	−0.000276
Unknown	5434 (9.7%)	2359 (11%)	−0.00870	5569 (9.9%)	2195 (9.8%)	0.00102
Smoking						
Current smoking	22 763 (41%)	10 073 (45%)		23 489 (42%)	9335 (42%)	
Former smoking	33 296 (59%)	12 246 (55%)	0.0452	32 562 (58%)	12 989 (58%)	−0.000910
Index of multiple deprivation						
1	7178 (13%)	3048 (14%)	−0.00846	7310 (13%)	2891 (13%)	0.000934
2	9213 (16%)	3813 (17%)	−0.00649	9321 (17%)	3730 (17%)	−0.000787
3	9971 (18%)	4077 (18%)	−0.00480	10 051 (18%)	4022 (18%)	−0.000835
4	12 722 (23%)	5012 (22%)	0.00235	12 680 (23%)	5061 (23%)	−0.000502
5	16 945 (30%)	6357 (28%)	0.0174	16 659 (30%)	6609 (30%)	0.00114
Missing	30 (< 0.1%)	12 (< 0.1%)	−2.44E‐06	29 (< 0.1%)	11 (< 0.1%)	5.40E‐05
Diabetes	14 081 (25%)	5517 (25%)	0.00397	14 021 (25%)	5617 (25%)	−0.00146
Hypertension	28 603 (51%)	11 319 (51%)	0.00311	28 563 (51%)	11 415 (51%)	−0.00173
Cardiovascular disease	16 772 (30%)	6540 (29%)	0.00618	16 682 (30%)	6665 (30%)	−0.000911
Cancer	10 543 (19%)	4416 (20%)	−0.00974	10 697 (19%)	4270 (19%)	−0.000435
Past asthma	15 346 (27%)	2665 (12%)	0.154	12 875 (23%)	5141 (23%)	−0.000602
Kidney impairment	16 724 (30%)	6738 (30%)	−0.00352	16 800 (30%)	6758 (30%)	−0.00300
Immunosuppression	665 (1.2%)	277 (1.2%)	−0.000547	674 (1.2%)	268 (1.2%)	1.33E‐06
Influenza vaccine	44 905 (80%)	17 962 (80%)	−0.00373	44 954 (80%)	17 898 (80%)	0.000252
Pneumococcal vaccine	5985 (11%)	3237 (15%)	−0.0382	6598 (12%)	2636 (12%)	−0.000376
COPD exacerbation in past 12 months	22 809 (41%)	6222 (28%)	0.128	20 759 (37%)	8290 (37%)	−0.00100

Abbreviations: BMI, body mass index; ICS, inhaled corticosteroid; LABA; long‐acting β‐agonist; SD, standard deviation; SMD; standardised mean difference.

#### 3.1.1 Association Between ICS and Clinical Outcomes

**FIGURE 1:**
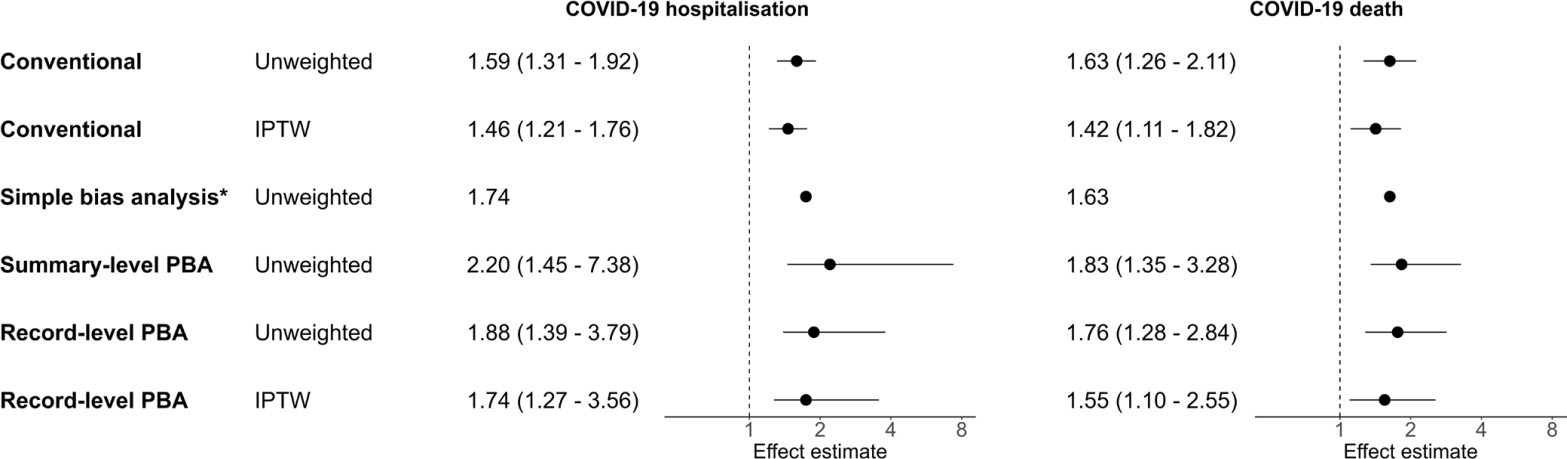
Forest plot of odds ratios and 95% confidence or simulation intervals for COVID‐19 hospitalisations and deaths, comparing ICS/LABA (± LAMA) users to LABA/LAMA users. Effect estimates > 1 indicate an increased risk in the ICS group. Conventional analysis refers to logistic regression without quantitative bias analysis. IPTW = inverse probability of treatment weighting; PBA = probabilistic bias analysis. *Simple bias analysis only results in a point estimate.

There were 662 COVID‐19 hospitalisations and 366 COVID‐19 deaths (Table 3). In unadjusted models, ICS users were at increased risk of all outcomes compared with LABA/LAMA users (OR for COVID‐19 hospitalisation 1.59 (95% CI 1.31–1.92); OR for COVID‐19 death 1.63 (95% CI 1.26–2.11)) (Figure 1). After IPTW, ORs shifted towards the null (OR for COVID‐19 hospitalisation 1.46 (95% CI 1.21–1.76); OR for COVID‐19 death 1.42 (95% CI 1.11–1.82)). We also observed an increased risk of all‐cause death among ICS users (OR 1.38 (95% CI 1.26–1.52)), which attenuated towards the null after IPTW (OR 1.23 (95% CI (1.12–1.34))) (results not shown).

**TABLE 3:** Observed outcomes by treatment group.

	ICS *N* = 56 059	LABA/LAMA *N* = 22 319
COVID‐19 hospitalisation	529 (0.9%)	133 (0.6%)
COVID‐19 death	294 (0.5%)	72 (0.3%)
All‐cause mortality	1969 (3.5%)	574 (2.6%)

#### 3.1.2 QBA Results for Hospitalisations

SBA resulted in an OR 1.75. Summary‐level PBA shifted ORs away from the null (OR 2.20 (95% SI 1.45–7.38)). Record‐level PBA with logistic regression gave a median OR of 1.88 (95% SI 1.39–3.79) and an IPT–weighted OR of 1.74 (95% SI 1.27–3.56). No iterations were discarded due to negative cell counts.

#### 3.1.3 QBA Results for Deaths

SBA for outcome misclassification of deaths gave an OR of 1.63. Summary‐level PBA of deaths resulted in an OR 1.83 (95% SI 1.35–3.28). Record‐level PBA with logistic regression gave a median unweighted OR of 1.76 (95% SI 1.28–2.84) and IPT–weighted OR of 1.55 (95% SI 1.10–2.55). 0.13% of iterations were discarded due to negative cell counts.

#### 3.1.4 Analysis Excluding People Using Triple Therapy

**FIGURE 2:**
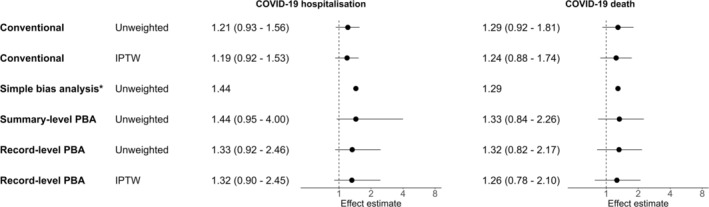
Forest plot of odds ratios and 95% confidence or simulation intervals for COVID‐19 hospitalisations and deaths, comparing ICS/LABA users to LABA/LAMA users, excluding triple therapy users. Effect estimates > 1 indicate an increased risk in the ICS group. Conventional analysis refers to logistic regression without quantitative bias analysis. IPTW = inverse probability of treatment weighting; PBA = probabilistic bias analysis. *Simple bias analysis only results in a point estimate.

Excluding people using triple therapy gave ORs 1.21 (95% CI 0.93–1.56) for COVID‐19 hospitalisations and 1.29 (95% CI 0.92–1.81) for COVID‐19 deaths (Figure 2). IPTW shifted the results towards the null (OR 1.19 (95% CI 0.92–1.53) for COVID‐19 hospitalisations, OR 1.24 (95% CI 0.88–1.74) for COVID‐19 deaths). Summary‐level QBA for COVID‐19 hospitalisations resulted in an OR of 1.44 (95% SI 0.95–4.00). Record‐level PBA with subsequent logistic regression resulted in an unweighted OR of 1.88 (95% SI 0.82–2.17) and a IPT–weighted OR of 1.26 (95% SI 0.78–2.10).

Summary‐level QBA for COVID‐19 deaths resulted in an OR of 1.33 (95% SI 0.84–2.26). Record‐level PBA with logistic regression resulted in an unweighted OR of 1.32 (95% SI 0.82–2.17) and an IPT–weighted OR of 1.26 (95% SI 0.78–2.10).

#### 3.1.5 Sensitivity Analyses

Analyses applying individual values for sensitivity and specificity showed that results were relatively insensitive to variations in sensitivity estimates, but small deviations from perfect specificity resulted in relatively marked shifts in the point estimate (e.g., for COVID‐19 hospitalisation, with perfect sensitivity, a decrease in specificity from 0.99 to 0.98 changed the median OR from 1.93 to 2.79; Figures S36, S37).

Simulation of differential sensitivity for the outcome COVID‐19 deaths showed that results were relatively robust to different values of sensitivity in the two treatment groups (Figures S38, S39). Results of Cox regression models were very similar to logistic regression models (Figures S40, S41).

## 4 Discussion

Among ICS users, the risk of COVID‐19 hospitalisation or death was higher than among LABA/LAMA users. Effect estimates attenuated towards the null after IPTW and after excluding people using triple therapy. This is likely explained by the fact that triple therapy users were in the ICS group and tend to have more severe COPD and comorbidities than LABA/LAMA users. Accounting for outcome misclassification using QBA shifted the effect estimates away from the null and increased the uncertainty around the point estimates, but did not change the conclusions of the analyses.

### 4.1 Comparison to Other Studies

Two previous studies used UK EHR data and the ONS Death Registry to investigate the same research question. The effect estimate in the OpenSAFELY study (aHR for COVID‐19 death 1.39 (1.10–1.76)) is very similar to ours, possibly due to similarities in study design [1]. A study using QResearch [4] found a more modest risk of severe COVID‐19 and COVID‐19 death associated with ICS use, independent of underlying respiratory disease (COVID‐19 hospitalisation aHR 1.13 (1.03–1.23); COVID‐19 death aHR 1.15 (1.01–1.31)). Both studies addressed confounding as a potential source of bias and discussed potential exposure misclassification. Several other studies investigated ICS and COVID‐19 outcomes, but in different populations, making results less comparable [2, 3, 35]. Despite concerns about COVID‐19 outcome misclassification, we are not aware of previous studies attempting to quantitatively correct for such misclassification.

### 4.2 Correcting for Potential Outcome Misclassification

All implementations of QBA shifted the effect estimates away from the null, in line with the heuristic that non‐differential misclassification biases results towards the null. This suggests conventional estimates underestimate the association between ICS and severe COVID‐19. However, our assessment adds confirmation that quantitatively accounting for plausible misclassification would not have led to different conclusions. Additionally, the uncertainty around the point estimates increased in PBA because the SI takes into account more sources of random error than the conventional CI [29]. As expected, QBA methods that additionally allowed confounding control gave ORs closer to the null. In sensitivity analyses looking at differential misclassification, we found that differential misclassification would have had to be substantial, with sensitivity of > 90% in the ICS group and < 60% in the LABA/LAMA group, to have concealed a protective association. We also found that even relatively small decreases in specificity resulted in substantial shifts in the effect estimate, while changes in sensitivity were less consequential.

The different forms of QBA used here [29, 36] have various strengths and limitations. SBA is easily implemented using available tools [37], but requires bias parameters to be known with relative certainty and does not estimate uncertainty of the results. PBA, while more analytically and computationally complex than SBA, offers several advantages, including the ability to incorporate uncertainty regarding the values of bias parameters. The SI generated during PBA incorporates systematic error arising from the modelled bias, error arising from the random nature of the misclassification process, uncertainty in the bias parameters and conventional random error. The 95% SI represents the 2.5th and 97.5th percentiles of the effect estimate distribution generated by PBA. Although incorporating QBA usually decreases the precision of the results, this may more accurately reflect the true total uncertainty of the result compared to the CI of the conventional analysis that only accounts for random error and neglects systematic error [29].

Both SBA and summary‐level PBA can be applied to a researcher's own or another researcher's work but are limited in their analysis, as the calculation of the effect estimates is based on 2 × 2 tables. Record‐level PBA is more sophisticated, as each data row is corrected for misclassification. Therefore, the analyst has complete analytical flexibility and can simultaneously account for confounding or varying follow‐up time. However, record‐level PBA requires access to the full dataset and substantial computational power, which may limit its application in large datasets. All methods of QBA performed similarly in this case study, but ultimately, the choice of QBA method depends on the degree of concern about the severity and impact of outcome misclassification, the analytical and computational resources available and the level of access to complete study data.

### 4.3 Strengths and Limitations

Strengths of this study include the size of CPRD Aurum and comprehensive capture of hospitalisations and deaths.

Due to the short follow‐up, we could not use a new‐user design and assumed that any effect of ICS on the outcomes was independent of treatment history and length. Therefore, the study population is heterogeneous regarding disease stage and treatment history. Covariates did not include symptoms of COPD or measures of lung function, which could have further controlled for confounding by disease severity. The analysis excluding triple therapy users may have been underpowered to detect increased risks in the ICS users, but it suggests that their inclusion led to confounding, highlighting the trade‐off between power and comparability of the treatment groups.

We did not conduct the analysis restricting to individual ICS due to low power. There may be differences in the effects of individual ICS, as there was some evidence that budesonide had a protective effect on COVID‐19 [11, 12], but this was not observed for ciclesonide or fluticasone [39, 40, 41].

Although outcome misclassification was thought to be the most impactful source of misclassification in this study, exposure misclassification may also have affected results. Previous work shows a spike in prescriptions in the United Kingdom in March 2020, meaning that estimated exposure durations may be less reliable than in non‐pandemic times [38]. Additionally, this study did not capture in‐hospital prescriptions.

Our approach to QBA required assumptions, which are untestable and likely imperfect. However, the alternative of not doing QBA assumes outcome sensitivity and specificity were both 100%, which is a less plausible position.

We did not investigate potential misclassification varying by a confounder. A confounder that may have resulted in differential misclassification is care home residence, but this data is not available in the United Kingdom. Simulation of differential misclassification with respect to exposure indicated that outcome classification would have needed to be very different in the exposure groups to change the study conclusions. Misclassification varying by a confounder would have had to be even more extreme to cause differential misclassification at the level of the exposure.

Early in the pandemic, treatments and the healthcare system's response to COVID‐19 were rapidly evolving, and it is likely that the accuracy of COVID‐19 outcome recording changed over time. The values and distributions chosen in this analysis represent best estimates. For excess deaths, chosen values were based on the strong assumption that all excess deaths were COVID‐19 deaths [31, 32]. Values for excess deaths were based on data from March to May 2020 [31], and sensitivity likely increased with time. For hospitalisations, estimates were based on validation studies in North America. While coding practices between countries and healthcare systems may differ, studies from the United Kingdom were not available, so these values were taken as the best available estimates.

## 5 Conclusions

We observed increased risks of COVID‐19 hospitalisation and death among ICS users compared to LABA/LAMA users. However, taken together with the results of the analysis excluding triple therapy users, the observed risk increase may be attributed to residual confounding, even after IPTW. QBA showed that accounting for plausible outcome misclassification would not have led to different study conclusions.

### 5.1 Plain Language Summary

During the COVID‐19 pandemic, researchers were concerned that some COVID‐19 cases might have been missed or misrecorded, which could affect studies looking at how treatments worked. This study looked at whether errors in recording COVID‐19 hospitalisations and deaths affected the results of studies investigating the link between inhaled corticosteroids (ICS) and COVID‐19 outcomes in people with chronic obstructive pulmonary disease (COPD) in the United Kingdom. We wanted to see if people with COPD using ICS were more likely to be hospitalised or die from COVID‐19, compared with people using other inhalers. We used a method called probabilistic bias analysis (PBA) to adjust for potential errors in how COVID‐19 hospitalisations and deaths were recorded. This method allows us to estimate how much incorrect or missing information might have changed our results. We found that people using ICS were more likely to be hospitalised or die from COVID‐19 than those using other inhalers, but the results suggested that the impact of incorrect recording was small and did not change the overall findings. However, there are still some concerns that other factors, like the reason why people were prescribed certain medications, might have influenced the results.

## Author Contributions

M.B., A.S., C.T.R. and I.D. contributed to the study design. M.B. conducted the data management and analysis and drafted the manuscript. All authors were involved in the design and conceptual development, and reviewed, edited and approved the final manuscript.

## Ethics Statement

The study was approved by the London School of Hygiene and Tropical Medicine Research Ethics Committee (Reference Number: 27896) and the Independent Scientific Advisory Committee of the UK Medicines and Healthcare Products Regulatory Agency (Approval Number: 22_001876).

## Conflicts of Interest

M.B. is funded by a GSK PhD studentship to investigate the application of quantitative bias analysis in observational studies of COVID‐19. I.D. has unrestricted grants from and shares in GSK. A.S. is employed by LSHTM on a fellowship funded by GSK. J.H. was employed by GSK when this work was initiated and owned stock in GSK during the conduct of this work, and is now an employee at Boehringer Ingelheim. C.T.R. and J.K.Q. report no conflicts of interest.

## Supporting information


**Data S1.** Supporting Information.

## Data Availability

No additional data is available. Data management and analysis code, along with all code lists, are available on our GitHub repository (https://github.com/bokern/ics_covid).

## References

[1] A. Schultze , A. J. Walker , B. MacKenna , et al., “Risk of COVID‐19‐Related Death Among Patients With Chronic Obstructive Pulmonary Disease or Asthma Prescribed Inhaled Corticosteroids: An Observational Cohort Study Using the OpenSAFELY Platform,” Lancet Respiratory Medicine 8 (2020): 1106–1120, 10.1016/S2213-2600(20)30415-X. 32979987 PMC7515601

[2] A. Husby , A. Pottegård , and A. Hviid , “Association Between Inhaled Corticosteroid Use and COVID‐19 Outcomes,” Pharmacoepidemiology and Drug Safety 30 (2021): 1486–1492, 10.1002/pds.5345. 34390285 PMC8441753

[3] C. I. Bloom , T. M. Drake , A. B. Docherty , et al., “Risk of Adverse Outcomes in Patients With Underlying Respiratory Conditions Admitted to Hospital With COVID‐19: A National, Multicentre Prospective Cohort Study Using the ISARIC WHO Clinical Characterisation Protocol UK,” Lancet Respiratory Medicine 9 (2021): 699–711, 10.1016/S2213-2600(21)00013-8. 33676593 PMC8241313

[4] P. Aveyard , M. Gao , N. Lindson , et al., “Association Between Pre‐Existing Respiratory Disease and Its Treatment, and Severe COVID‐19: A Population Cohort Study,” Lancet Respiratory Medicine 9 (2021): 909–923, 10.1016/S2213-2600(21)00095-3. 33812494 PMC8016404

[5] World Health Organization , “Emergency Use ICD Codes for COVID‐19 Disease Outbreak,” accessed July 3, 2023, https://www.who.int/standards/classifications/classification‐of‐diseases/emergency‐use‐icd‐codes‐for‐covid‐19‐disease‐outbreak.

[6] S. Kung , M. Doppen , M. Black , et al., “Underestimation of COVID‐19 Mortality During the Pandemic,” ERJ Open Research 7 (2021): 00766–2020, 10.1183/23120541.00766-2020. 33614772 PMC7734715

[7] Global Initiative for Asthma , “Global Strategy for Asthma Management and Prevention (2021 Update),” (2021).

[8] D. M. G. Halpin , D. Singh , and R. M. Hadfield , “Inhaled Corticosteroids and COVID‐19: A Systematic Review and Clinical Perspective,” European Respiratory Journal 55 (2020): 2001009, 10.1183/13993003.01009-2020. 32341100 PMC7236828

[9] D. M. G. Halpin , R. Faner , O. Sibila , J. R. Badia , and A. Agusti , “Do Chronic Respiratory Diseases or Their Treatment Affect the Risk of SARS‐CoV‐2 Infection?,” Lancet Respiratory Medicine 8 (2020): 436–438, 10.1016/S2213-2600(20)30167-3. 32251625 PMC7270536

[10] Y. Jamilloux , T. Henry , A. Belot , et al., “Should We Stimulate or Suppress Immune Responses in COVID‐19?,” Cytokine and Anti‐Cytokine Interventions. Autoimmunity Reviews 19 (2020): 102567, 10.1016/J.AUTREV.2020.102567. 32376392 PMC7196557

[11] L.‐M. Yu , M. Bafadhel , J. Dorward , et al., “Inhaled Budesonide for COVID‐19 in People at High Risk of Complications in the Community in the UK (PRINCIPLE): A Randomised, Controlled, Open‐Label, Adaptive Platform Trial,” Lancet 398 (2021): 843–855, 10.1016/S0140-6736(21)01744-X. 34388395 PMC8354567

[12] S. Ramakrishnan , D. V. Nicolau , B. Langford , et al., “Inhaled Budesonide in the Treatment of Early COVID‐19 (STOIC): A Phase 2, Open‐Label, Randomised Controlled Trial,” Lancet Respiratory Medicine 9 (2021): 763–772, 10.1016/S2213-2600(21)00160-0. 33844996 PMC8040526

[13] S. M. Langan , S. A. Schmidt , K. Wing , et al., “The Reporting of Studies Conducted Using Observational Routinely Collected Health Data Statement for Pharmacoepidemiology (RECORD‐PE),” BMJ 363 (2018): k3532, 10.1136/bmj.k3532. 30429167 PMC6234471

[14] Clinical Practice Research Datalink , “CPRD Aurum March 2022,” (2022), 10.48329/my9s-4x08.

[15] A. Wolf , D. Dedman , J. Campbell , et al., “Data Resource Profile: Clinical Practice Research Datalink (CPRD) Aurum,” International Journal of Epidemiology 48 (2019): 1740–1740g, 10.1093/IJE/DYZ034. 30859197 PMC6929522

[16] “Hospital Episode Statistics (HES) Admitted Patient Care and CPRD Primary Care Data Documentation (Set 21),” (2021).

[17] Clinical Practice Research Datalink , “CPRD Linked Data|CPRD,” accessed December 9, 2021, https://www.cprd.com/linked‐data#HES%20Admitted%20Patient%20Care%20data.

[18] “ONS Death Registration Data and CPRD Primary Care Data Documentation (Set 21),” (2021).

[19] J. K. Quint , H. Müllerova , R. L. DiSantostefano , et al., “Validation of Chronic Obstructive Pulmonary Disease Recording in the Clinical Practice Research Datalink (CPRD‐GOLD),” BMJ Open 4 (2014): e005540, 10.1136/BMJOPEN-2014-005540/-/DC1. PMC4120321 25056980

[20] S. Schneeweiss , J. A. Rassen , J. S. Brown , et al., “Graphical Depiction of Longitudinal Study Designs in Health Care Databases,” (2019), 10.7326/M18-3079. 30856654

[21] K. J. Rothnie , H. Müllerová , J. R. Hurst , et al., “Validation of the Recording of Acute Exacerbations of COPD in UK Primary Care Electronic Healthcare Records,” PLoS One 11 (2016): e0151357, 10.1371/JOURNAL.PONE.0151357. 26959820 PMC4784784

[22] J. A. C. Sterne , I. R. White , J. B. Carlin , et al., “Multiple Imputation for Missing Data in Epidemiological and Clinical Research: Potential and Pitfalls,” BMJ 338 (2009): b2393, 10.1136/bmj.b2393. 19564179 PMC2714692

[23] M. M. Garrido , A. S. Kelley , J. Paris , et al., “Methods for Constructing and Assessing Propensity Scores,” Health Services Research 49 (2014): 1701–1720, 10.1111/1475-6773.12182. 24779867 PMC4213057

[24] J. N. Hunnicutt , C. M. Ulbricht , S. A. Chrysanthopoulou , and K. L. Lapane , “Probabilistic Bias Analysis in Pharmacoepidemiology and Comparative Effectiveness Research: A Systematic Review,” Pharmacoepidemiology and Drug Safety 25 (2016): 1343–1353, 10.1002/pds.4076. 27593968 PMC5272921

[25] M. P. Fox , R. F. MacLehose , and T. L. Lash , “SAS and R Code for Probabilistic Quantitative Bias Analysis for Misclassified Binary Variables and Binary Unmeasured Confounders,” International Journal of Epidemiology 52 (2023): 1624–1633, 10.1093/ije/dyad053. 37141446 PMC10555728

[26] “Applying Quantitative Bias Analysis to Epidemiologic Data,” accessed January 13, 2024, https://sites.google.com/site/biasanalysis/.

[27] S. S. Kadri , J. Gundrum , S. Warner , et al., “Uptake and Accuracy of the Diagnosis Code for COVID‐19 Among US Hospitalizations,” JAMA 324 (2020): 2553–2554, 10.1001/jama.2020.20323. 33351033 PMC7756233

[28] G. Wu , A. G. D'Souza , H. Quan , et al., “Validity of ICD‐10 Codes for COVID‐19 Patients With Hospital Admissions or ED Visits in Canada: A Retrospective Cohort Study,” BMJ Open 12 (2022): e057838, 10.1136/bmjopen-2021-057838. PMC8787827 35063962

[29] M. P. Fox , R. F. MacLehose , and T. L. Lash , Applying Quantitative Bias Analysis to Epidemiologic Data (Cham: Springer International Publishing, 2021), 10.1007/978-3-030-82673-4.

[30] S. A. Kluberg , L. Hou , S. K. Dutcher , et al., “Validation of Diagnosis Codes to Identify Hospitalized COVID‐19 Patients in Health Care Claims Data,” Pharmacoepidemiology and Drug Safety 31 (2022): 476–480, 10.1002/pds.5401. 34913208

[31] “Analysis of Death Registrations Not Involving Coronavirus (COVID‐19), England and Wales—Office for National Statistics,” accessed May 17, 2023, https://www.ons.gov.uk/peoplepopulationandcommunity/birthsdeathsandmarriages/deaths/articles/analysisofdeathregistrationsnotinvolvingcoronaviruscovid19englandandwales28december2019to1may2020/technicalannex.

[32] “Deaths in England|Coronavirus in the UK,” https://coronavirus.data.gov.uk/details/deaths?areaType=nation&areaName=England (2023).

[33] StataCorp LLC , “Stata Statistical Software: Release 17 College Station,” (2021).

[34] Team RCR , “A Language and Environment for Statistical Computing,” R Foundation for Statistical Computing (2024).

[35] J. C. Choi , S.‐Y. Jung , U. A. Yoon , et al., “Inhaled Corticosteroids and COVID‐19 Risk and Mortality: A Nationwide Cohort Study,” Journal of Clinical Medicine 9 (2020): 3406, 10.3390/jcm9113406. 33114246 PMC7690894

[36] H. R. Banack , E. Hayes‐Larson , and E. R. Mayeda , “Monte Carlo Simulation Approaches for Quantitative Bias Analysis: A Tutorial,” Epidemiologic Reviews 43 (2021): 106–117, 10.1093/epirev/mxab012. PMC9005059 34664653

[37] “Bias.Analysis,” accessed June 17, 2024, https://sites.google.com/site/biasanalysis/Home.

[38] J. S. Frazer and G. R. Frazer , “Analysis of Primary Care Prescription Trends in England During the COVID‐19 Pandemic Compared Against a Predictive Model,” Family Medicine and Community Health 9 (2021): e001143, 10.1136/fmch-2021-001143. 34344766 PMC8338320

[39] N. Ezer , S. Belga , N. Daneman , et al., “Inhaled and Intranasal Ciclesonide for the Treatment of Covid‐19 in Adult Outpatients: CONTAIN Phase II Randomised Controlled Trial,” BMJ 375 (2021): e068060, 10.1136/bmj-2021-068060. 34728476 PMC8561042

[40] B. M. Clemency , R. Varughese , Y. Gonzalez‐Rojas , et al., “Efficacy of Inhaled Ciclesonide for Outpatient Treatment of Adolescents and Adults With Symptomatic COVID‐19: A Randomized Clinical Trial,” JAMA Internal Medicine (2021), 10.1001/JAMAINTERNMED.2021.6759. PMC8609464 34807241

[41] D. R. Boulware , C. J. Lindsell , T. G. Stewart , et al., “Inhaled Fluticasone Furoate for Outpatient Treatment of Covid‐19,” New England Journal of Medicine 389 (2023): 1085–1095, 10.1056/NEJMoa2209421. 37733308 PMC10597427

